# Assessment of the insecticidal activity of oral afoxolaner against *Phlebotomus perniciosus* in dogs

**DOI:** 10.1051/parasite/2019063

**Published:** 2019-11-05

**Authors:** Nadège Perier, Wilfried Lebon, Leon Meyer, Noua Lekouch, Nesrine Aouiche, Frédéric Beugnet

**Affiliations:** 1 Boehringer Ingelheim Animal Health 29 avenue Tony Garnier 69007 Lyon France; 2 Clinvet Douar Dbabej Beni Yekhlef 28815 Mohammedia Morocco

**Keywords:** *Phlebotomus perniciosus*, Sandfly, Insecticide, Afoxolaner, NexGard^®^, Dog

## Abstract

Twelve healthy dogs were included in this laboratory efficacy study. Six dogs were randomly allocated based on body weight to an untreated control group and six to an afoxolaner (NexGard^®^) treated group. In the treatment group, afoxolaner was administered orally on Day 0 in accordance with label instructions. On Days 1, 14 and 28, each dog was exposed to 60 unfed female and 10 male *Phlebotomus perniciosus* sandflies for 1 h. At the end of each exposure period, sandflies were counted and assessed for viability and feeding status. There was no statistical difference in mortality (0.0–5.4%), nor in feeding proportion (61.6–78%) between the control and the treated groups at all 1-h post-exposure assessments. After collection, live fed and unfed sandflies were kept for viability assessments at 48 and 72 h post-exposure. In the untreated control group, the average percentages of live, fed, female sandflies after exposure, on Days 1, 14 and 28, ranged from 51% to 74% at 48 h and from 46% to 57% at 72 h, demonstrating model robustness over the 28 days of the study. Significantly fewer live fed sandflies were recorded for the afoxolaner treated group (*p* < 0.01). The insecticidal efficacy was 100%, 95.9% and 75.2% at 48 h post Days 1, 14 and 28 exposures, respectively, and 100%, 100% and 86.3% at 72 h post Days 1, 14, and 28 exposures, respectively. A single administration of oral afoxolaner (NexGard^®^) to dogs significantly killed *P. perniciosus* sandflies 48 and 72 h after blood feeding for 1 month.

## Abstract (fr)

Douze chiens en bonne santé ont été inclus dans cette étude d’efficacité en laboratoire. Six chiens ont été répartis au hasard en fonction de leur poids corporel dans un groupe témoin non traité et six dans un groupe traité par afoxolaner (NexGard^®^), administré par voie orale le jour 0 conformément aux instructions de l’étiquette. Les jours 1, 14 et 28, chaque chien a été exposé à 60 femelles à jeun et 10 mâles de *Phlebotomus perniciosus* pendant une heure. À la fin de chaque période d’exposition, les phlébotomes ont été évalués en termes de viabilité et de statut alimentaire. Il n’y avait pas de différence statistique dans la mortalité (0,0 à 5,4 %), ni dans le taux d’engorgement (61,6 à 78 %) entre le groupe témoin et le groupe traité lors de toutes les évaluations après une heure. Après la collecte, les phlébotomes vivants gorgés et non gorgés ont été conservés aux fins d’évaluation de la viabilité 48 et 72 heures après l’exposition. Dans le groupe témoin non traité, le pourcentage moyen de phlébotomes femelles gorgées et vivantes après l’exposition aux jours 1, 14 et 28 variait de 51 à 74 % à 48 heures et de 46 à 57 % à 72 heures, démontrant la robustesse du modèle au cours des 28 jours de l’étude. Un nombre significativement moins important de phlébotomes gorgés vivants ont été enregistrés dans le groupe traité par afoxolaner (*p* < 0,01). L’efficacité insecticide était de 100 %, 95,9 % et 75,2 % 48 heures après les expositions des jours 1, 14 et 28, respectivement, et 100 %, 100 % et 86,3 % à 72 heures après les expositions des jours 1, 14 et 28, respectivement. Une seule administration d’afoxolaner (NexGard^®^) par voie orale à un chien tue de manière significative les phlébotomes *P. perniciosus* 48 heures et 72 heures après la prise de sang pendant un mois.

## Introduction

Canine leishmaniosis (CL) is an infectious disease due to the proliferation of the protozoan flagellate parasite *Leishmania infantum* in cells of the reticulo-endothelial system (i.e., monocyte cell line) [7]. This parasite is mainly transmitted by the bite of phlebotomine sandflies (*Phlebotomus* in the Old World, i.e., Africa, Asia, Europe; and *Lutzomyia* in the Americas) [11, 15, 16, 26]. *Leishmania* protozoans can also be transmitted, rarely, by blood transfusion, or vertically from mothers to their puppies [28].

Although dogs constitute the main reservoir, *L. infantum* can also infect many other mammals like lagomorphs, rodents, foxes, cats, horses, and humans [15, 16, 26, 28]. CL is a major zoonosis and human cases are reported in endemic areas where the prevalence of CL in dogs is high [16, 28]. Two hundred cases in Italy and approximately 25 autochthonous cases in France are reported yearly [27, 30].

Canine leishmaniosis is endemic in more than 70 countries. It is highly endemic in countries around the Mediterranean basin, but also West Africa, Southern Asia, and Central and South America. In endemic countries, the seroprevalence in dogs can vary greatly from a few percentage points to more than 50%. Distribution can be highly heterogeneous between endemic foci (i.e., high seroprevalence in dogs, multiple clinical cases) and ectopic or new foci (i.e., low prevalence, few clinical cases and no or very few vectors) [15, 16, 26, 27].

Recent surveys have demonstrated a gradual spread to previously non-infected areas [10, 26, 27, 30]. Several authors have described new outbreaks from Southern or Central Italy to Northern Italy, such as Tuscany, Marche and Emilia-Romagna; in France with a spread to the West and Northwest [10]; as well as Catalonia, in northeastern Spain [1, 23], and Galicia, in northern Spain [25]. Canine leishmaniosis is becoming endemic in the Balkans and Romania, with extension towards Central and Northern Europe [23].

*Phlebotomus perniciosus* is one of the major vectors of canine leishmaniosis in Southern Europe [19, 20]. Other *Phlebotomus* species are also involved in North Africa, Southeastern Europe and Central Asia, e.g. *Phlebotomus ariasi, P. perfiliewi, P. sergenti*. *P. perniciosus* is a ubiquitous sandfly living in urban and peri-urban areas and having crepuscular activity [20]. *P. perniciosus* is a proven vector of this protozoan in Algeria, France, Italy, Malta, Portugal, Spain, Greece, and Turkey, and is a suspected vector in Morocco and Tunisia [24]. Over the past decades, there has been an increase in sandfly geographical distribution and density, which can be attributed to climate and ecological changes, but also to increased tourism [23, 24, 26].

There are two essential strategies to limit the transmission of *L. infantum* to dogs and humans: (1) control of the canine reservoir by using insecticides with repellent activity to prevent sandfly bites, by treating infected dogs, and by vaccinating dogs in enzootic areas; (2) control and reduce the vector density by acting on sandflies and/or sandfly ecosystems [26, 30].

Female sandflies take their blood meal in a short time, approximately 4 min and are able to inoculate *Leishmania* during that time [2, 9]. Therefore, systemic insecticides will not prevent the infection of dogs during the sandfly bite. However, they may decrease the population of sandflies and avoid further bites and transmission to mammals because sandflies take 6–10 days to lay eggs before biting a new host again [13]. They also do not fly long distances (maximum 1 km) and usually stay concentrated around 200–500 m [19, 20].

Afoxolaner is a systemic insecticide and acaricide compound from the isoxazoline group. Afoxolaner acts by inhibition of a specific receptor on GABA-gated chloride ion channels, resulting in uncontrolled activity of the central nervous system and death of the arthropods [28]. After oral administration, afoxolaner is rapidly absorbed and is highly bound to plasma proteins, therefore acting through a systemic pathway on hematophagous arthropods [21]. Afoxolaner is available as a palatable chew [18] given orally at the minimum dose of 2.5 mg/kg (NexGard^®^, Boehringer Ingelheim Animal Health). It is indicated for the treatment and control of fleas and ticks in dogs [3, 4, 12, 17], the treatment of *Demodex* and *Sarcoptes* [5, 6]*,* and has been proven effective against *Otodectes* mites [8]*.* More recently, the insecticidal activity of afoxolaner against *Aedes aegypti* mosquitoes at 24 h post-exposure was demonstrated. It showed that *A. aegypti* mosquitoes ingested a lethal dose of afoxolaner during their blood meal [22]. Based on these results, we hypothesized that afoxolaner could also kill sandflies that feed on afoxolaner treated dogs [13].

Afoxolaner has no repellent properties. Nevertheless, we hypothesized that it could be a good candidate in the control of sandfly populations by killing those that would feed on treated dogs. Killing female sandflies before a new bite and preventing them from laying eggs would help to reduce the sandfly population in a restricted area.

## Materials and methods

The design and conditions of this study were approved by animal welfare ethical committees of Boehringer-Ingelheim and ClinVet, and were performed in accordance with International Cooperation on Harmonization of Technical Requirements for Registration of Veterinary Medicinal Products (VICH) Guideline 9, entitled Good Clinical Practice. The containment of the dogs also complied with the Directive 2010/63/EU of the European Parliament and of the Council of 22 September 2010 on the protection of animals used for scientific purposes, and was approved by the Institutional Animal Care and Use Committee (IACUC). This study was a parallel group, blinded, randomized, negative controlled efficacy study. It was conducted with two groups of six dogs each.

A total of 12 healthy laboratory Beagle dogs (six males and six females) between 14 and 18 months-old and weighing between 7.9 and 13.6 kg were included in the study. Dogs were acclimatized to the study conditions for seven days and examined by a veterinarian. The 12 dogs were randomly allocated to two groups of six dogs (untreated control group and afoxolaner treated group) based on body weight. All dogs were observed daily from acclimation start to the end of the study for general health. None of the dogs participating in this study had been treated with any acaricide/insecticide compound within three months preceding Day 0. The animals were kept individually in cages with visual and auditory contact with conspecifics. Dogs from the same group were allowed to access an outdoor shared exercise area from Day 7 onwards. At least one toy was made available to each dog (replenished weekly). The dog cages were part of a semi-indoor animal unit with a natural photoperiod.

On Day 0, NexGard^®^ (2.27% w/w afoxolaner chewable tablets) was administered orally to all dogs assigned to the treated group in accordance with European label instructions. On Day 1, 14 and 28, dogs were exposed to 60 unfed females and 10 males *P. perniciosus*, in a dark room, in a dark room for 60 min (±5 min). The sandfly numbers varied for each dog and each challenge, but the exact counts were performed at collection time 1 h after each exposure. For the sandfly exposure, each dog was sedated using medetomidine (Domitor^®^, Zoetis) and the head of the dog was placed into a sandfly proof net (dimensions: 40 cm × 40 cm × 40 cm). Although males do not take blood meals, their presence improves female engorgement [28]. At the end of each 1-h exposure period, sandflies were smoothly vacuumed from the enclosure, categorized (male/female), counted and assessed for viability status (live/dead). As classically performed for insects and acarians, moribund sandflies were counted as live, which is more restrictive in the assessment of effectiveness [22]. Sandflies were also categorized as either fed or unfed, separated, and transferred to containers. All live fed female sandflies were then kept and incubated in vials at approximately 25 °C and >60% relative humidity to perform further viability assessments (live, dead) at 48 and 72 h after each exposure.

A laboratory-bred strain of *P. perniciosus* originating from Italy was used for the exposures [29]. Sandflies were unfed and aged from 3 to 10 days on the day of challenge.

The efficacy of afoxolaner against *P. perniciosus* was calculated using the total number of live fed female sandflies at 1, 48 and 72 h after each exposure, according to the formula below:

$$\mathrm{Insecticidal}\;\mathrm{efficacy}\;(\%)\;\mathrm{against}\;\mathrm{sandflies}=100\times (P_{c}-P_{t})P_{c},$$

where *P*_*c*_ = Arithmetic mean number of the proportion* of live fed female sandflies in the control group; *P*_*t*_ = Arithmetic mean number of the proportion* of live fed female sandflies in the treated group;

$$*\mathrm{Proportion}\;\mathrm{of}\;\mathrm{live}\;\mathrm{fed}\;\mathrm{sandflies}\;\mathrm{per}\;\mathrm{animal}=\left[\left(\mathrm{Live}\;\mathrm{fed}\;\mathrm{sandflies}\left(\mathrm{Live}+\mathrm{Dead}\right)\mathrm{fed}\;\mathrm{sandflies}\right)\right].$$

In addition, feeding proportion (at each 1-h post-exposure) and mortality % (1, 48 and 72 h post-exposure) were calculated for each control dog, according to the formulas below:

$$\begin{array}{c} \mathrm{Feeding}\;\mathrm{proportion}\;\left[\%\right]=\left[\left(\mathrm{Total}\;\mathrm{fed}\;\mathrm{sandflies}\mathrm{Total}\;\mathrm{collected}\;\mathrm{sandflies}\right)\times 100\right],\\ \mathrm{Mortality}\;\left[\%\right]=\left[\left(\mathrm{Total}\;\mathrm{dead}\;\mathrm{fed}\;\mathrm{sandflies}\mathrm{Total}\;\mathrm{collected}\;\mathrm{fed}\;\mathrm{sandflies}\right)\times 100\right]. \end{array}$$

The groups were compared using a Wilcoxon Sum Rank Test. SAS Version 9.3 TS Level 1M2 was used for the statistical analyses.

## Results

No adverse event was recorded after treatment or during the study duration.

The live or dead status, as well as the engorgement status, of the sandflies was assessed at all time-points (Table 1). The mortality observed in the control group on Days 1, 14 and 28 after the 1-h exposure ranged from 0.0% to 3.3% (Table 2) and the feeding proportion ranged from 66% to 77% (Table 3), indicating that the sandfly strain was vigorous and that the feeding model worked. The mortality observed in the treated group on Days 1, 14 and 28 after the 1-h exposure ranged from 0.0% to 5.4% (Table 2) and the feeding proportion ranged from 61.6% to 78% (Table 3). There was no statistical difference in the mortality rate, nor in the feeding proportion between the control and the treated groups at all 1-h post-exposure assessments for all time-points.

**Table 1:** Arithmetic means (±95% confidence interval [CI], *α* = 0.05) for female sandflies status at 1, 48, and 72 h post-exposure.

Group	Hours post-exposure	Days of exposure and status of collected female sandflies											
		Day 1				Day 14				Day 28			
		Fed live	Fed dead	Unfed live	Unfed dead	Fed live	Fed dead	Unfed live	Unfed dead	Fed live	Fed dead	Unfed live	Unfed dead
Control	1 h	37.8 (±8.5)	0 (na)	13.8 (±6.9)	0.2 (±0.3)	26.7 (±7.3)	1.0 (±0.9)	6.7 (±3.3)	1.5 (±1.9)	35.8 (±13.6)	0.5 (±0.7)	15.0 (±9.0)	1.5 (±1.4)
	48 h	24.5 (±5.2)	13.3 (±7.0)	12.0 (±6.1)	2.0 (±1.2)	15.0 (±7.3)	12.7 (±2.3)	4.7 (±3.1)	3.5 (±2.5)	26.3 (±9.4)	10.0 (±5.4)	11.3 (±7.7)	5.2 (±4.7)
	72 h	20.7 (±5.1)	17.2 (±7.9)	10.3 (±5.3)	3.7 (±1.9)	14.0 (±6.6)	13.7 (±2.2)	3.8 (±2.5)	4.3 (±2.2)	20.5 (±6.6)	15.8 (±9.0)	9.5 (±5.8)	7.0 (±5.4)
Afoxolaner	1 h	38.2 (±5.0)	0 (na)	9.5 (±4.9)	1.8 (±2.3)	26.5 (±7.0)	1.2 (±1.2)	8.8 (±5.8)	3.3 (±1.6)	34.7 (±12.0)	0.7 (±0.4)	16.3 (±10.4)	4.8 (±2.9)
	48 h	0 (na)	38.2 (±5.0)	5.0 (±3.5)	5.3 (±3.9)	0.7 (±0.7)	27.0 (±5.9)	6.0 (±5.6)	6.2 (±2.7)	7.7 (±10.6)	27.7 (±11.8)	10.7 (±7.8)	10.5 (±3.6)
	72 h	0 (na)	38.2 (±5.0)	4.0 (±2.4)	6.3 (±4.7)	0 (na)	27.7 (±6.3)	5.3 (±4.4)	6.8 (±3.0)	4.0 (±7.1)	31.3 (±10.6)	8.0 (±5.0)	13.2 (±5.4)

na, not applicable.

**Table 2:** Mortality % of fed female sandflies at 1, 48, and 72 h post-exposure.

Group	Day	Hours post-exposure		
		1 h	48 h	72 h
		Mortality (%)	Mortality (%)	Mortality (%)
Control	D1	0.0	33.3	43.5
	D14	3.3	49.3	52.5
	D28	0.9	26.3	39.5
Afoxolaner	D1	0.0	100.0	100.0
	D14	5.4	97.9	100.0
	D28	2.6	81.8	91.7

Mortality [%] = [(Total dead fed sandflies/Total collected fed sandflies) × 100].

**Table 3:** Feeding proportion of female sandflies at 1 h post-exposure.

Group	Day	Feeding proportion (%)
Control	D1	72.8
	D14	77.0
	D28	66.0
Afoxolaner	D1	78.0
	D14	69.6
	D28	61.6

Feeding proportion [%] = [(Total fed sandflies/Total collected sandflies) × 100].

In the control group, sandfly mortality was 33.3%, 49.3% and 26.3% at 48 h and 43.5%, 52.5% and 39.5% at 72 h post-exposure on Days 1, 14 and 28, respectively. In the treated group, 100% of the fed sandflies were dead at the 48 h assessment on Day 1, 100% at 72 h on Day 14 and 91.7% at 72 h on Day 28 (Table 2).

Significantly fewer live fed sandflies were recorded for the afoxolaner treated group compared to the negative control group, both at 48 and 72 h assessments, after each exposure on Days 1, 14 and 28 (*p* < 0.01) (Table 4).

**Table 4:** Average proportions of live fed sandflies and insecticidal efficacy at 1, 48 and 72 h post-exposure.

Day	Hours post-exposure	Average proportion of live fed sandflies ± 95% CI		Insecticidal efficacy (%)	*p*-value**
		Control group	Afoxolaner group		
1	1 h	100*	100*	–	
	48 h	66.7 ± 13.6	0*	100%	*p* < 0.0027
	72 h	56.5 ± 13.1	0*	100%	*p* < 0.0027
14	1 h	96.7 ± 2.5	94.6 ± 6.0	–	
	48 h	50.7 ± 213.6	2.1 ± 1.9	95.9%	*p* < 0.0047
	72 h	47.5 ± 311.8	0*	100%	*p* < 0.0047
28	1 h	99.1 ± 1.2	97.4 ± 2.1	–	
	48 h	73.7 ± 6.6	18.2 ± 21.2	75.2%	*p* < 0.0129
	72 h	60.5 ± 12.2	8.3 ± 14.4	86.3%	*p* < 0.0043

Proportion of live fed sandflies per animal [%] = [(Live fed sandflies/Total fed sandflies) × 100].

Insecticidal efficacy (%) against sandflies = 100 × (*P*_*c*_−*P*_*t*_)/*P*_*c*_, where *P*_*c*_ = Arithmetic mean number of the proportion of live fed female sandflies in the control group; *P*_*t*_ = Arithmetic mean number of the proportion of live fed female sandflies in the treated group.

* For all samples showing 0 or 100% live sandflies, there is no 95% CI.

** *p*-value based on a Wilcoxon sum rank test on live fed female counts between groups at each time-point.

The afoxolaner insecticidal efficacy against *P. perniciosus* was 100%, 95.9% and 75.2% at 48 h post day 1, 14 and 28 challenges, and 100%, 100% and 86.3% at 72 h post day 1, 14, and 28 challenges (Table 4).

## Discussion and conclusions

The study demonstrated the insecticidal activity, thus the mortality of female sandflies, after a blood meal on afoxolaner treated dogs. Female sandflies take their blood meal in a short time, approximately 4 min [9], and they ingest 4–5 μL of blood [2, 11]. Proteins provided by the blood meal are necessary for egg production [19, 20]. *P. perniciosus* eggs are laid 6–10 days after a blood meal, and before the next blood meal [29]. With a limited volume of blood and therefore a low quantity of afoxolaner ingested, we did not expect an immediate killing effect, but did expect the death within several hours or days after feeding, so before the females lay eggs or bite a second time. *Leishmania* promastigotes need 7 to 10 days to become infective in the female sandfly [11, 19]. Therefore treating dogs having canine leishmaniosis would prevent transmission to other dogs. *Phlebotomus* sandflies do not fly long distances (maximum 1 km) and stay usually concentrated around 200–500 m. Therefore, the biological features are in favor of the possibility of obtaining a decrease in the sandfly population biting afoxolaner treated dogs, thereby possibly reducing the rate of *Leishmania* transmission in endemic areas [13].

In this study, the observed mortality of sandflies in the control group at 1 h post exposure was low (less than 3.3% after each challenge) and the feeding proportion was higher than 66%, demonstrating the relevance of the experimental model.

A single administration of afoxolaner was enough to kill 100% of sandflies within 48 h of exposure on Day 1, 96% and 100% of sandflies within 48 h and 72 h of exposure on Day 14; and 75.0 and 86.4% of sandflies within 48 h and 72 h of exposure on Day 28, respectively. Recently another isoxazoline, fluralaner, also demonstrated efficacy against *Phlebotomus papatasi* sandflies after oral administration to dogs [14]. The authors reached a similar conclusion on the beneficial activity of systemic isoxazolines for the control of vector populations.

Isoxazolines, like afoxolaner or fluralaner, do not provide prevention of *Leishmania* transmission as the female sandflies bite before dying. Therefore, preventative measures like repellent application or vaccination are still needed for individual dog protection. Nevertheless, the insecticidal activity of systemically active isoxazolines against the vector is an additional beneficial measure to decrease vector density and decrease risk at the population level.

## Competing interest

The work reported herein was funded by Boehringer-Ingelheim Animal Health. Wilfried Lebon and Frédéric Beugnet are current employees of Boehringer-Ingelheim. Nadège Perier and Nesrine Aouiche are veterinarians, finishing a Master 2 degree in Science at Lyon University. Leon Meyer and Noua Lekouch are employees of the CRO ClinVet.

NEXGARD^®^ is a registered trademark of Merial, now part of Boehringer-Ingelheim. All other marks are the property of their respective owners.

This document is provided for scientific purposes only. Any reference to a brand or trademark herein is for informational purposes only and is not intended for a commercial purpose or to dilute the rights of the respective owner(s) of the brand(s) and trademark(s).
